# Frailty and Incident Depressive Symptoms During Short- and Long-Term Follow-Up Period in the Middle-Aged and Elderly: Findings From the Chinese Nationwide Cohort Study

**DOI:** 10.3389/fpsyt.2022.848849

**Published:** 2022-04-07

**Authors:** Haiyang Liu, Xu Yang, Lei-lei Guo, Jin-long Li, Guang Xu, Yunxiao Lei, Xiaoping Li, Lu Sun, Liu Yang, Ting Yuan, Congzhi Wang, Dongmei Zhang, Huanhuan Wei, Jing Li, Mingming Liu, Ying Hua, Yuanzhen Li, Hengying Che, Lin Zhang

**Affiliations:** ^1^Student Health Center, Wannan Medical College, Wuhu, China; ^2^Department of Radiotherapy, The First Affiliated Hospital of Jinzhou Medical University, Jinzhou, China; ^3^Department of Surgical Nursing, School of Nursing, Jinzhou Medical University, Jinzhou, China; ^4^Department of Occupational and Environmental Health, Key Laboratory of Occupational Health and Safety for Coal Industry in Hebei Province, School of Public Health, North China University of Science and Technology, Tangshan, China; ^5^Department of Radiotherapy, Third Affiliated Hospital of Jinzhou Medical University, Jinzhou, China; ^6^Obstetrics and Gynecology Nursing, School of Nursing, Wannan Medical College, Wuhu, China; ^7^Department of Emergency and Critical Care Nursing, School of Nursing, Wannan Medical College, Wuhu, China; ^8^Department of Internal Medicine Nursing, School of Nursing, Wannan Medical College, Wuhu, China; ^9^Department of Pediatric Nursing, School of Nursing, Wannan Medical College, Wuhu, China; ^10^Department of Surgical Nursing, School of Nursing, Wannan Medical College, Wuhu, China; ^11^Rehabilitation Nursing, School of Nursing, Wannan Medical College, Wuhu, China; ^12^Department of Nursing, Yijishan Hospital, The First Affiliated Hospital of Wannan Medical College, Wuhu, China

**Keywords:** cohort study, depressive symptoms, frailty, incidence, middle-aged and elderly

## Abstract

**Objective:**

Prefrailty and frailty are two common conditions among older individuals. Recent studies have reported the association between frailty and depressive symptoms, but whether those conditions could predict depressive symptoms is still inconsistent in a few longitudinal studies. In our study, we aimed to estimate the cross-sectional and longitudinal associations between frailty and incident depressive symptoms in a nationally representative sample of community-dwelling middle-aged and older Chinese adults.

**Method:**

Data were obtained from the China Health and Retirement Longitudinal Study (CHARLS), which included 17,284 adults aged ≥ 45 years. Participants were followed every two years using a face-to-face, computer-aided personal interview (CAPI) and structured questionnaire. We excluded participants with no follow-up data. The numbers of individuals who completed the baseline surveys were 2,579 and follow-up surveys were 839 for the short-term (2 years from 2011 to 2013) and 788 for the long-term (4 years from 2011 to 2015). In addition, Frailty was measured by the Fried criteria and depressive symptoms were estimated by the Chinese version of the Center for Epidemiologic Studies-Depression scale (CES-D). Logistic regression was used to analyze the odds ratio (OR), and 95% confidence interval (CI) for the cross-sectional associations of frailty and its components with depressive symptoms in the participants at baseline. Cox proportional hazards analysis was conducted using the hazard ratio (HR), and 95% confidence interval (CI) for the prospective associations of baseline frailty and pre-frailty and its component in the participants without depressive symptoms at baseline.

**Results:**

At baseline, 57.93% of participants had depressive symptoms and 55.84% had pre-frail and 11.63% had frailty. In the cross-sectional analysis, both pre-frailty (OR = 5.293, 95%CI 4.363–6.422) and frailty (OR = 16.025, 95%CI 10.948–23.457) were associated with depressive symptoms. In the longitudinal analysis, frailty [HR = 1.395 (0.966–2.013)] and pre-frailty [HR = 2.458 (0.933, 6.479)] were not significantly associated with incident depressive symptoms in a full-adjusted model among participants free of baseline depressive symptoms during the short-term. However, frailty [HR = 1.397 (1.017, 1.920)] and pre-frailty [HR = 2.992 (1.210, 7.397)] were significantly associated with incident depressive symptoms during the short term. In the components of frailty, slowness [HR = 1.597 (1.078, 2.366)] was associated with an increased risk of depressive symptoms onset during the short-term. Weakness [HR = 2.08 (1.055, 4.104)] and exhaustion [HR = 1.928 (1.297, 2.867)] were associated with increased risk of depressive symptoms onset during the short-term.

**Conclusion:**

Among the middle-aged and older adults, frailty, pre-frailty did not predict depressive symptoms during 2 years of follow-up, when accounting for the potential confounders, slowness considered alone predicted depressive symptoms. Additionally, frailty, pre-frailty predicted depressive symptoms during 4 years of follow-up, when accounting for the potential confounders, weakness and exhaustion considered alone predicted depressive symptoms.

## Introduction

The depressive symptom is a common medical disorder condition among middle-aged and elderly adults with a prevalence of 37.84% in China (1). Depressive symptom severity is related to serious results such as sadness, disability, increasing the burden of patients, family, and society (2, 3). Frailty, defined by a clinical syndrome of increased vulnerability to stressors, is characterized by loss of physiologic reserve among older age, which is associated with disability, hospitalization, and death (4). The most well-known biological syndrome model of frailty is characterized as exhaustion, weakness, low physical activity, slowness, and weight loss (pre-frailty as the presence of one or two of these components, and frailty as the presence of three or more components) (5). Most studies included adults aged ≥65years, the proportions of 10.7% for frailty and 41.6% for prefrailty (6). A meta-analysis including 240 studies reported that the prevalence of frailty was 11-13% among the aged ≥50 adults, and the prefrailty was 45–48% (7). Cross-sectional analysis of 18,227 selected community-dwelling individuals 50 years of age and older in Europe in 2004 found that prevalence of frailty was 4.1%, and prefrail was 37.4% (8). 2006–2010 Bus Santé study including participants aged 50 and more showed that 28.7% and 7.8% presented one frailty indicator, and two or more frailty indicators, respectively (9). Other studies reported that the prevalence of frailty was 61.6% in the middle-aged diabetic population (10), and 6.1% in HIV-infected individuals.

Mounting evidence demonstrates that frailty could be associated with depression. The current meta-analysis sought to estimate the strength of the relationship between frailty and depression. A recent meta-analysis conducted with fourteen studies (10 cohort studies and 4 cross-sectional studies) by Chu et al. (11) found that older adults aged 65 years or older, with depression, are more prone to frailty than are those without depression (OR = 2.99, 95%CI 2.19–4.08). Furthermore, older males with depression were at a higher risk for frailty than older females with depression (female: OR = 2.25, 95%CI 1.54–3.27; male: OR = 4.76, 95%CI 3.61–6.27). Another meta-analysis performed for the 24 studies by Soysal et al. (12) found that (I) people with depression were at increased odds of having frailty (OR = 4.07, 95%CI 1.93–8.55), and (II) older adults with frailty were at increased odds of having depression (OR = 2.64, 95%CI: 1.59–4.37), and (III) frailty at the baseline increased the risk of incident frailty by about 272% (HR = 3.72, 95%CI 1.95–7.08); and (IV) frailty at the baseline increased the risk of incident depression by about 90% (HR = 1.90, 95%CI 1.55–2.32). In addition, the other three meta-analysis studies (13–15) also tested the association between frailty and depression and supported for a bidirectional association between depression and frailty. Although the meta-analysis included cohort studies and cross-sectional studies and highlighted the potential adverse effect of frailty on depression, no considerations of the components effect of frailty on depression over several years in different studies were made. Moreover, the meta-analysis only included Western participants, and no considerations of other ethnicities, such as Asians. Thus, further studies among the middle-aged and elderly in Asian nations are needed to identify whether the relationship between frailty and incidence of depression in Asian participants.

To address these gaps, we used 4 years of longitudinal data from the nationally-representative sample of community-dwelling Chinese participants aged ≥45 years and explored to examine the relationship between frailty and incidence of depressive symptoms during the short- (2 years) and long-term (4 years) internals. Furthermore, our study explores the stability of the association between components of frailty and depressive symptoms by controlling potential confounders.

## Materials and Methods

### Study Participants

We obtained the data from The China Health and Retirement Longitudinal Study (CHARLS). The CHARLS began in 2011 with a cohort of 17,284 participants ≥45 years (Wave1). Subsequently, data collection was conducted in 2013 (Waves2), 2015 (Waves3). CHARLS was a nationally representative longitudinal survey of the mid-aged and elderly population in China along with their spouses. The respondents will be followed every 2 years using a face-to-face, computer-aided personal interview (CAPI) and structured questionnaire. The current study used data from participants who participated in Wave1, Waves2, and Waves3. We excluded individuals who met any of the following criteria at baseline (1) Chinese version of the Center for Epidemiologic Studies-Depression scale (CESD-10) ≥10 scores, (2) no components of frailty data, (3) no age/sex/educational levels/marital status/current smoking/alcohol drinking/exercise/chronic diseases/live place/ activities data. In addition, we excluded participants with no follow-up data. The numbers of individuals who completed both the baseline were 2,579 individuals, and follow-up surveys were 839 for the short-term (2 years from 2011 to 2013), and 788 for the long-term (4 years from 2011 to 2015).

### Depressive Symptom

The Chinese version of the Center for Epidemiologic Studies-Depression scale (CES-D) (16–19) was used to measure depressive symptoms in the study. The CES-D consists of 10 items, and each item uses a 4-point Likert scale [from 0 “Rarely or none of the time (<1 day)” to 3 “Most or all of the time (5–7 days)”] to reflect the severity of a particular symptom during the past week. The total score ranges from 0 to 30, with a higher total score indicating more severe depressive symptoms. We used harmonized criteria cutoff values to define depressive symptom-total score ≥10. The Chinese version of the CES-D has shown adequate reliability and validity in the middle-aged and elderly. In our study, the Cronbach alpha coefficient was 0.86 and the construct validity was 0.62 (1).

### Frailty Assessment

Frailty was evaluated through the widely used criteria originally proposed by Fried et al. (5), modified with the information available in the CHARLS. In the definition, frailty contained five components of exhaustion, weakness, low physical activity, weight loss, and slowness. In our study, the five components of frailty were evaluated and defined as follows: (1) **Exhaustion:** exhaust was present if the participant answered “Most or all of the time” or “Occasionally or a moderate amount of the time” for either of the Chinese version of the Center for Epidemiologic Studies-Depression scale (CES-D) questions (20): “I felt everything I did was an effort during last week” or “I could not get going during last week.” This component was constructed identically to the originally proposed by Fried et al. (5). (2) **Weakness:** weakness was measured using the self-reported item “having difficulty in Lifting or carrying weights over 10 jin, like a heavy bag of groceries” (21). (3) **Low physical activity:** CHARLS defined WALK as walking those participants might do solely for recreation, sport, excise, or leisure at work and at home, walking to travel from place to place, and any other walking. Low physical activity was considered to be present if the participants had no physical activity or WALK at least 10 min at a time during a usual week. This component was different from that proposed by Fried et al. (5), but similar treatment variables have been used before to evaluate the low physical activity (22). (4) **Weight loss:** the weight loss was defined as the unintentional loss of 5 or more kilograms in the last year (21) or current body mass index (BMI) ≤ 18.5 kg/m^2^ (23). (5) **Slowness:** these questions asked the participants to report whether they have difficulty with walking 100 meters or climbing several flights of stairs without resting, which was similar to that used in other studies (21). Those participants who had difficulty with performing the walking or climbing were categorized as slowness. In our study, robustness was defined as the absence of any component, pre-frailty as the presence of one or two of these components, and frailty as the presence of three or more components.

### Body Measurement

Body measure index (BMI) was defined as the body mass (kg) divided by the square of the body height (m). BMI were divided into 4 categories: underweight (BMI < 18.5 kg/m^2^), normal (18.5–24 kg/m^2^), overweight (24–28 kg/m^2^), and obese (≥28 kg/m^2^) (24–26).

### Covariates

Age, sex (male, female), educational levels, marital status, current residence, current smoking, alcohol drinking, chronic diseases, activities at baseline, and entry wave (Wave 1, 2, 3) were incorporated as covariates in the present research. (1) Age was classified into four categories: below 45–54, 55–64, 65–74, and above 75 yr old. (2) Educational levels including illiterate (no formal education), less than elementary school (did not finish primary school but capable of reading or writing, graduate from sishu/home school, elementary school, or middle school), high school, and above vocational school (graduate from two/three-year college/associate degree, graduate from post-graduate, doctoral degree/Ph. D). (3) Marital status was categorized into two groups: the single (divorced, and never married, widowed, or separated) and married. (4) Current residence including rural and urban. (5) Current smoking including current smoker, former-smoker and never smoked. (6) Alcohol drinking including never drinker, less than once a month, and more than once a month. (7) Activities (interacted with friends/ provided help to family, friends, or neighbors who do not live with you and who did not pay you for the help/went to a sport, social, or other kind of club/played Ma-jong, played chess, played cards, or went to community club/took part in a community-related organization/done voluntary or charity work/cared for a sick or disabled adult who does not live with you and who did not pay you for the help/attended an educational or training course/stock investment /used the Internet) were dichotomized as ever (at least once a month) vs. never. (8) Chronic diseases, including (1) hypertension, (2) cancer or malignant tumor (excluding minor skin cancers), (3) diabetes or high blood sugar, (4) chronic lung diseases, (5) dyslipidemia, (6) liver disease (except fatty liver, tumors, and cancer), (7) kidney disease (except for tumor or cancer), (8) stroke, (9) heart attack, coronary heart disease, angina, congestive heart failure, or other heart problems, (10) stomach or other digestive disease (except for tumor or cancer), (11) memory-related disease, (12) emotional, nervous, or psychiatric problems, (13) arthritis or rheumatism, (14) asthma were reported by the respondents (diagnosed by a doctor). According to previous standard (1, 27), a continuous variable is used to reflect the presence of chronic health conditions among 14 common chronic diseases which ranges from 0 to 14. The numbers of the condition of the chronic disease were classified into three categories: 0, 1–2, and 3–14. The categories have been widely used in our previous studies (1, 24–26, 28–31).

### Statistical Analysis

Statistical analyses were performed using the IBM SPSS version25.0 (IBM Corp., Armonk, NY). Categorical variables were expressed as frequencies and percentages and compared using the χ^2^ test. Logistic regression was used to analyze the odds ratio (OR), and 95% confidence interval (CI) for the cross-sectional associations of frailty and its components with depressive symptoms in the participants at baseline. Depressive symptoms were analyzed as binary dependent variable (no-depressive symptoms and depressive symptoms), and covariates were sequentially entered into the regression models. Model 1 included frailty or its components only, model 2 additionally included socio-demographic characteristics (age, sex, educational levels, marital status, current residence), and model 3 further included health behaviors and condition (current smoking, alcohol drinking, activities, chronic diseases), and model 4 additionally included body measure (BMI). Cox proportional hazards analysis was conducted using the hazard ratio (HR), and 95% confidence interval (CI) for the prospective associations of baseline frailty and pre-frailty and its component in the participants without depressive symptoms at baseline. Covariates were modeled by methods identical to those in cross-sectional analysis. A two-sided *P*-value of <0.05 was considered a statistically significant level.

## Results

Table 1 shows the baseline characteristics of participants according to the level of depressive symptoms. The mean age of participants was 61.06 (SD ± 10.12); 38.31% were male; 16.01% were single; 91.74% were living in rural. 9.00% were former smoking, and 24.58% were current smoking; 7.10% were drinking less than once a month, and 19.35% were drinking more than once a month; 49.24% were taking activities; 51.38% had 1–2 chronic diseases, and 27.14% had 3–14 chronic diseases. The frequency of depressive symptoms was 57.93%. The differences among participants with or without depressive symptoms were observed in the distribution of age subgroups, sex, educational levels, marital status, living place, cigarette smoking, alcohol consumption status, and chronic diseases.

**Table 1:** Baseline characteristics of participants according to the level of depressive symptoms in CHARLS Waves 2011 (*N*, %).

**Variables**	**All** **participants (2,579)**	**No-depressive symptoms** **(1,085)**	**Depressive symptoms** **(1,494)**	***t*/χ^2^**	***P*-value**
Age (years)	61.06 ± 10.12	61.67 ± 10.29	60.61 ± 9.98	2.623	0.006
Age groups (years)					
45–54	727 (28.19)	298 (27.47)	429 (28.71)	10.074	0.018
55–64	948 (36.76)	370 (34.10)	578 (38.69)		
65–74	620 (24.04)	287 (26.45)	333 (22.29)		
≥75	284 (11.01)	130 (11.98)	154 (10.31)		
Sex					
Male	988 (38.31)	499 (45.99)	489 (32.73)	46.761	0.000
Female	1,591 (61.69)	586 (54.01)	1,005 (67.27)		
Education					
Illiterate	890 (34.51)	328 (30.23)	562 (37.62)	44.975	0.000
Less than elementary school	1,517 (58.82)	647 (59.63)	870 (58.23)		
High school	102 (3.96)	62 (5.71)	40 (2.68)		
Above vocational school	70 (2.71)	48 (4.42)	22 (1.47)		
Marital status					
Single	413 (16.01)	153 (14.1)	260 (17.40)	5.094	0.024
Married	2,166 (83.99)	932 (85.9)	1,234 (82.60)		
Current residence					
Rural	2,366 (91.74)	973 (89.68)	1,393 (93.24)	10.526	0.001
Urban	213 (8.26)	112 (10.32)	101 (6.76)		
Current smoking					
No	1,713 (66.42)	675 (62.21)	1,038 (69.48)	16.487	0.000
Former smoke	232 (9.00)	118 (10.88)	114 (7.63)		
Current smoke	634 (24.58)	292 (26.91)	342 (22.89)		
**Alcohol drinking**					
No	1,897 (73.56)	771 (71.06)	1,126 (75.37)	9.220	0.010
Less than once a month	183 (7.10)	74 (6.82)	109 (7.30)		
More than once a month	499 (19.35)	240 (22.12)	259 (17.34)		
Taking activities					
No	1,309 (50.76)	528 (48.66)	781 (52.28)	3.281	0.070
Yes	1,270 (49.24)	557 (51.34)	713 (47.72)		
Chronic diseases (counts)	1.76 ± 1.49	1.55 ± 1.42	1.92 ± 1.53	1.932	0.000
Chronic diseases groups (counts)					
0	554 (21.48)	282 (25.99)	272 (18.21)	42.020	0.000
1–2	1,325 (51.38)	572 (52.72)	753 (50.40)		
3–14	700 (27.14)	231 (21.29)	469 (31.39)		
BMI (kg/m^2^)					
BMI categories	23.70 ± 4.09	23.86 ± 4.08	23.58 ± 4.09	0.214	0.078
<18.5	200 (7.75)	68 (6.27)	132 (8.84)	6.808	0.078
18.5–24	1,253 (48.58)	530 (48.85)	723 (48.39)		
24–28	789 (30.59)	348 (32.07)	441 (29.52)		
≥28	337 (13.07)	139 (12.81)	198 (13.25)		

Table 2 shows the baseline characteristics of participants according to the level of frailty. A total of 2,579 robust individuals (32.53%), pre-frail (55.84%), and frailty (11.63%) at baseline were included in the cross-sectional analysis. The differences among components of frailty were observed in the distribution of age subgroups, sex, educational levels, marital status, living place, alcohol consumption status, taking activities, chronic diseases, and BMI categories.

**Table 2:** Baseline characteristics of participants according to the level of frailty in CHARLS Waves2011.

**Variables**	**All** **participants (2,579)**	**Robust (839)**	**Pre-frail (1,440)**	**Frailty (300)**	***F*/χ^2^**	***P*-value**
Age (years)	61.06 ± 10.12	60.11 ± 9.84	60.61 ± 9.88	65.83 ± 10.79	39.506	0.000
Age groups (years)						
45–54	727 (28.19)	263 (31.35)	425 (29.51)	39 (13)	81.216	0.000
55–64	948 (36.76)	317 (37.78)	528 (36.67)	103 (34.33)		
65–74	620 (24.04)	185 (22.05)	347 (24.1)	88 (29.33)		
≥75	284 (11.01)	74 (8.82)	140 (9.72)	70 (23.33)		
Sex						
Male	988 (38.31)	347 (41.36)	556 (38.61)	85 (28.33)	15.990	0.000
Female	1,591 (61.69)	492 (58.64)	884 (61.39)	215 (71.67)		
Education						
Illiterate	890 (34.51)	245 (29.2)	494 (34.31)	151 (50.33)	71.495	0.000
Less than elementary school	1,517 (58.82)	505 (60.19)	872 (60.56)	140 (46.67)		
High school	102 (3.96)	47 (5.6)	49 (3.4)	6 (2)		
Above vocational school	70 (2.71)	42 (5.01)	25 (1.74)	3 (1)		
Marital status						
Single	413 (16.01)	108 (12.87)	240 (16.67)	65 (21.67)	13.740	0.001
Married	2,166 (83.99)	731(87.13)	1,200 (83.33)	235 (78.33)		
Current residence						
Rural	2,366 (91.74)	745 (88.8)	1,338 (92.92)	283 (94.33)	14.890	0.001
Urban	213 (8.26)	94 (11.2)	102 (7.08)	17 (5.67)		
Current smoking						
No	1,713 (66.42)	565 (67.34)	932 (64.72)	216 (72)	9.004	0.061
Former smoke	232 (9)	77 (9.18)	127 (8.82)	28 (9.33)		
Current smoke	634 (24.58)	197 (23.48)	381 (26.46)	56 (18.67)		
Alcohol drinking						
No	1,897 (73.56)	594 (70.8)	1,058 (73.47)	245 (81.67)	16.428	0.002
Less than once a month	183 (7.1)	71 (8.46)	93 (6.46)	19 (6.33)		
More than once a month	499 (19.35)	174 (20.74)	289 (20.07)	36 (12)		
Taking activities						
No	1,309 (50.76)	361 (43.03)	769 (53.4)	179 (59.67)	33.617	0.000
Yes	1,270 (49.24)	478 (56.97)	671 (46.6)	121 (40.33)		
Chronic diseases (counts)	1.76 ± 1.49	1.43 ± 1.33	1.83 ± 1.47	2.38 ± 1.77	50.520	0.000
Chronic diseases categories						
0	554 (21.48)	229 (27.29)	287 (19.93)	38 (12.67)	80.684	0.000
1–2	1,325 (51.38)	457 (54.47)	732 (50.83)	136 (45.33)		
3–14	700 (27.14)	153 (18.24)	421 (29.24)	126 (42)		
BMI (kg/m^2^)	23.70 ± 4.09	24.00 ± 4.05	23.61 ± 4.00	23.26 ± 4.55	4.289	0.014
BMI categories						
<18.5	200 (7.75)	37 (4.41)	118 (8.19)	45 (15)	41.081	0.000
18.5–24	1,253 (48.58)	409 (48.75)	703 (48.82)	141 (47)		
24–28	789 (30.59)	284 (33.85)	434 (30.14)	71 (23.67)		
≥28	337 (13.07)	109 (12.99)	185 (12.85)	43 (14.33)		

Table 3 shows baseline characteristics classified according to the subsequent onset of depressive symptoms. In the short-term (2 years from 2011 to 2013), participants who developed depressive symptoms were more likely to be female and to live in rural. They tended to be never smoking. In the long-term (4 years from 2011 to 2015), participants who developed depressive symptoms were also more likely to be female and to take no activities.

**Table 3:** Baseline characteristics classified according to subsequent onset of depressive symptoms.

**Variables**	**2011 → 2013** **Incidence rate** **(*N* = 839,%)**	***P1***	**2011 → 2015** **Incidence rate** **(*N* = 788,%)**	***P2***
Age (years)		0.313		0.121
45–54	8.34		11.17	
55–64	8.82		11.93	
65–74	7.39		7.74	
≥75	1.67		1.27	
Sex		0.000		0.000
Male	9.42		11.04	
Female	16.81		21.07	
Education		0.204		0.245
Illiterate	8.46		10.41	
Less than elementary school	15.49		18.91	
High school	1.67		1.65	
Above vocational school	0.6		1.14	
Marital status		0.124		0.671
Single	23.6		28.43	
Married	2.62		3.68	
Current residence		0.025		0.853
Rural	24.67		29.06	
Urban	1.55		3.05	
Current smoking		0.004		0.136
No	18.59		21.95	
Former smoke	1.43		2.28	
Current smoke	6.2		7.87	
Alcohol drinking		0.258		0.081
No	19.55		24.62	
Less than once a month	1.31		2.03	
More than once a month	5.36		5.46	
Taking activities		0.590		0.008
No	12.75		17.51	
Yes	13.47		14.59	
Chronic diseases (counts)		0.126		0.752
0	5.6		8.5	
1–2	15.14		17.13	
3–14	5.48		6.47	
BMI (kg/m^2^)		0.525		
<18.5	1.79		1.78	0.344
18.5–24	13.71		16.12	
24–28	7.75		9.39	
≥28	2.98		4.82	

Table 4 shows the cross-sectional relationship between frailty and depressive symptoms at baseline. Both pre-frailty (OR = 5.293, 95%CI 4.363–6.422) and frailty (OR = 16.025, 95%CI 10.948–23.457) were significantly associated with depressive symptoms after adjusting for age, sex, educational levels, marital status, live place, current smoking, alcohol drinking, activities, chronic diseases, and BMI (adjusted model 4). In the frailty component, after adjusting for the full set of covariates, exhaust (OR = 12.094, 95%CI 9.864–14.827), weakness (OR = 2.058, 95%CI 1.526–2.775), weight loss (OR = 1.526, 95%CI 1.167–1.995), and slowness (OR = 1.849, 95%CI 1.521–2.247) were associated with prevalent depressive symptoms. However, low physical activity (OR = 1.227, 95%CI 0.973–1.548) was not associated with prevalent depressive symptoms.

**Table 4:** Odds ratios (ORs) and 95% confidence interval (CIs) for depressive symptoms at baseline associated with frailty and components of frailty at baseline.

***N* = 2,579**	**Model 1** **OR (95%CI)**	**Wald, df**	***P-*value**	**Model 2** **OR (95%CI)**	**Wald, df**	***P-*value**	**Model 3** **OR (95%CI)**	**Wald, df**	***P-*value**	**Model 4** **OR (95%CI)**	**Wald, df**	***P*-value**
Frailty status												
Robust (839)	Ref (1.000)			Ref (1.000)			Ref (1.000)			Ref (1.000)		
Pre-frail (1,440)	5.413 (4.493, 6.522)	315.891,1	0.000	5.539 (4.572, 6.710)	305.97,1	0.000	5.32 (4.385, 6.453)	287.728,1	0.000	5.293 (4.363, 6.422)	285.437,1	0.000
Frailty (300)	15.942 (11.07, 22.96)	221.346,1	0.000	17.445 (11.958, 25.45)	220.146,1	0.000	16.228 (11.091, 23.743)	205.974,1	0.000	16.025 (10.948, 23.457)	203.67,1	0.000
*P*-trend	4.696 (4.020, 5.487)	379.743,1	0.000	4.852 (4.131, 5.698)	370.504,1	0.000	4.668 (3.970, 5.489)	347.338,1	0.000	4.642 (3.947, 5.46)	343.942,1	0.000
Weakness												
No (2,301)	Ref (1.000)			Ref (1.000)			Ref (1.000)			Ref (1.000)		
Yes (278)	2.395 (1.802, 3.182)	36.251,1	0.000	2.266 (1.688, 3.043)	29.6,1	0.000	2.089 (1.55, 2.816)	23.426,1	0.000	2.058 (1.526, 2.775)	22.387,1	0.000
Slowness												
No (1,875)	Ref (1.000)			Ref (1.000)			Ref (1.000)			Ref (1.000)		
Yes (704)	1.969 (1.638, 2.367)	51.916,1	0.000	1.979 (1.634, 2.398)	48.715,1	0.000	1.833 (1.508, 2.227)	37.154,1	0.000	1.849 (1.521, 2.247)	38.066,1	0.000
Weight loss												
No (2,285)	Ref (1.000)			Ref (1.000)			Ref (1.000)			Ref (1.000)		
Yes (294)	1.688 (1.301, 2.189)	15.556,1	0.000	1.677 (1.287, 2.185)	14.669,1	0.000	1.575 (1.206, 2.058)	11.11,1	0.001	1.526 (1.167, 1.995)	9.533,1	0.002
Exhaustion												
No (1,360)	Ref (1.000)			Ref (1.000)			Ref (1.000)			Ref (1.000)		
Yes (1,219)	12.185 (10.014, 14.828)	623.315,1	0.000	12.511 (10.218, 15.319)	598.182,1	0.000	12.177 (9.934, 14.927)	578.921,1	0.000	12.094 (9.864, 14.827)	574.89,1	0.000
Low activity												
No (2,188)	Ref (1.000)			Ref (1.000)			Ref (1.000)			Ref (1.000)		
Yes (391)	1.169 (0.938, 1.457)	1.929,1	0.165	1.201 (0.955, 1.509)	2.458,1	0.117	1.208 (0.959, 1.523)	2.565,1	0.109	1.227 (0.973, 1.548)	2.994,1	0.084

*Model 1, unadjusted; Model 2, adjusted for age, sex, educational levels, marital status, live place; Model 3, adjusted for age, sex, educational levels, marital status, live place, current smoking, alcohol drinking, activities, chronic diseases; Model 4, adjusted for age, sex, educational levels, marital status, live place, current smoking, alcohol drinking, activities, chronic diseases, BMI*.

Table 5 shows the prospective associations between baseline frailty and depressive symptoms at 2- and 4-years follow-up survey in the participants without depressive symptoms at baseline. Firstly, in crude analysis, pre-frailty and frailty were not significantly associated with incident depressive symptoms during the short-term [pre-frail: HR = 1.316 (0.959, 1.805); frailty: HR = 2.162 (0.945, 4.948)]. Secondly, in crude analysis, the frailty [HR = 1.386 (1.019, 1.885)] and pre-frailty [HR = 2.492 (1.054, 5.893)] were significantly associated with incident depressive symptoms during the long-term. Thirdly, after adjusting for age, sex, educational levels, marital status, live place, current smoking, alcohol drinking, activities, chronic diseases, and BMI, frailty [HR = 1.395 (0.966, 2.013)] and pre-frailty [HR = 2.458 (0.933, 6.479)] were not significantly associated with incident depressive symptoms during the short-term. Lastly, after adjusting for the full set of covariates, the HR for pre-frailty was 1.397 (95%CI 1.017–1.920) and for frailty was 2.992 (95%CI 1.210–7.397) during the long-term.

**Table 5:** Association between frailty and incident depressive symptoms not depressed at baseline.

**Follow-up period**		**Model 1** **HR (95%CI)**	**Wald, df**	***P-*value**	**Model 2** **HR (95%CI)**	**Wald, df**	***P-*value**	**Model 3** **HR (95%CI)**	**Wald, df**	***P-*value**	**Model 4** **HR (95%CI)**	**Wald, df**	***P-*value**
**2011 → 2013**	Frailty status												
***N*** **= 839**	Robust (471)	Ref (1.000)			Ref (1.000)			Ref (1.000)			Ref (1.000)		
	Pre-frail (343)	1.316 (0.959, 1.805)	2.9,1	0.089	1.366 (0.991, 1.882)	3.624,1	0.057	1.380 (0.957, 1.989)	2.972,1	0.085	1.395 (0.966, 2.013)	3.155,1	0.076
	Frailty (25)	2.162 (0.945, 4.948)	3.332,1	0.068	2.207 (0.943, 5.168)	3.326,1	0.068	2.487 (0.948, 6.519)	3.431,1	0.064	2.458 (0.933, 6.479)	3.308,1	0.069
	*P*-trend	1.366 (1.041, 1.793)	5.046,1	0.025	1.404 (1.064, 1.853)	5.756,1	0.016	1.443 (1.053, 1.978)	5.2,1	0.023	1.451 (1.057, 1.990)	5.316,1	0.021
**2011 → 2015**	Frailty status												
***N*** **= 788**	Robust (447)	Ref (1.000)			Ref (1.000)			Ref (1.000)			Ref (1.000)		
	Pre-frail (319)	1.386 (1.019, 1.885)	4.33,1	0.037	1.450 (1.06, 1.983)	5.417,1	0.020	1.400 (1.018, 1.923)	4.295,1	0.038	1.397 (1.017, 1.920)	4.249,1	0.039
	Frailty (22)	2.492 (1.054,5.893)	4.326,1	0.038	2.939 (1.208, 7.153)	5.644,1	0.018	3.036 (1.231, 7.487)	5.815,1	0.016	2.992 (1.210, 7.397)	5.63,1	0.018
	*P*-trend	1.443 (1.103, 1.886)	7.178,1	0.007	1.525 (1.159, 2.006)	9.08,1	0.003	1.494 (1.130, 1.976)	7.94,1	0.005	1.489 (1.126, 1.969)	7.775,1	0.005

*Model 1, unadjusted; Model 2, adjusted for age, sex, educational levels, marital status, live place; Model 3, adjusted for age, sex, educational levels, marital status, live place, current smoking, alcohol drinking, activities, chronic diseases; Model 4, adjusted for age, sex, educational levels, marital status, live place, current smoking, alcohol drinking, activities, chronic diseases, BMI*.

Table 6 shows the association between components of frailty and incident depressive symptoms not depressed at baseline. Firstly, in crude analysis, depressive symptoms risk was increased for the slowness [HR = 1.519 (1.046, 2.204)] during the short-term. However, weakness, weight loss, exhaustion, and low activity were not significantly associated with incident depressive symptoms [weakness: HR = 1.334 (0.694, 2.566), weight loss: HR = 1.631 (0.983, 2.705), exhaustion [HR=1.410 (0.944, 2.104), low activity: HR = 0.972 (0.604, 1.563)]. Secondly, in crude analysis, depressive symptoms risk was increased for the weakness [HR = 2.205 (1.146, 4.243)] and exhaustion [HR = 1.853 (1.264, 2.716)] during the long-term. However, slowness, weight loss, and low activity were not significantly associated with incident depressive symptoms [slowness: HR = 1.006 (0.687, 1.474), weight loss: HR = 0.986 (0.574, 1.693), low activity: HR = 1.478 (0.941, 2.32)]. Thirdly, after adjusting for age, sex, educational levels, marital status, live place, current smoking, alcohol drinking, activities, chronic diseases, and BMI, the HR for slowness was 1.597 (95%CI 1.078, 2.366) during the short-term. However, weakness, weight loss, exhaustion, and low activity were not significantly associated with incident depressive symptoms [weakness: HR = 1.186 (0.602, 2.334), weight loss: HR = 1.510 (0.895, 2.548), exhaustion: HR = 1.353 (0.897, 2.041), low activity: HR = 1.023 (0.627, 1.668)]. Lastly, adjusting for the full set of covariates, the HR for weakness was 2.080 (95%CI 1.055, 4.104) and for exhaustion was 1.928 (95%CI 1.297, 2.867) during the long-term. However, slowness, weight loss, and low activity were not significantly associated with incident depressive symptoms [slowness: HR = 0.998 (0.670, 1.487), weight loss: HR = 0.992 (0.568, 1.735), low activity: HR = 1.582 (0.988, 2.533)].

**Table 6:** Association between components of frailty and incident depressive symptoms not depressed at baseline.

**Follow-up period**		**Model 1** **HR (95%CI)**	**Wald, df**	***P-*value**	**Model 2** **HR (95%CI)**	**Wald, df**	***P-*value**	**Model 3** **HR (95%CI)**	**Wald, df**	***P-*value**	**Model 4** **HR (95%CI)**	**Wald, df**	***P-*value**
**2011 → 2013**	Weakness												
***N*** **= 839**	No (795)	Ref (1.000)			Ref (1.000)			Ref (1.000)			Ref (1.000)		
	Yes (44)	1.334 (0.694, 2.566)	0.747,1	0.387	1.29 (0.662, 2.516)	0.56,1	0.454	1.224 (0.623, 2.403)	0.343,1	0.558	1.186 (0.602, 2.334)	0.243,1	0.622
	**Slowness**												
	No (679)	Ref (1.000)			Ref (1.000)			Ref (1.000)			Ref (1.000)		
	Yes (160)	1.519 (1.046, 2.204)	4.828,1	0.028	1.617 (1.101, 2.376)	5.995,1	0.014	1.549 (1.048, 2.288)	4.829,1	0.028	1.597 (1.078, 2.366)	5.454,1	0.020
	Weight loss												
	No (766)	Ref (1.000)			Ref (1.000)			Ref (1.000)			Ref (1.000)		
	Yes (73)	1.631 (0.983, 2.705)	3.592,1	0.058	1.632 (0.976, 2.73)	3.488,1	0.062	1.587 (0.944, 2.667)	3.042,1	0.081	1.510 (0.895, 2.548)	2.38,1	0.123
	**Exhaustion**												
	No (705)	Ref (1.000)			Ref (1.000)			Ref (1.000)			Ref (1.000)		
	Yes (134)	1.410 (0.944, 2.104)	2.82,1	0.093	1.394 (0.928, 2.093)	2.556,1	0.110	1.352 (0.897, 2.037)	2.079,1	0.149	1.353 (0.897, 2.041)	2.082,1	0.149
	**Low activity**												
	No (738)	Ref (1.000)			Ref (1.000)			Ref (1.000)			Ref (1.000)		
	Yes (101)	0.972 (0.604, 1.563)	0.014,1	0.907	1.006 (0.619, 1.634)	0.001,1	0.982	0.997 (0.612, 1.625)	0,1	0.991	1.023 (0.627, 1.668)	0.008,1	0.929
**2011 → 2015**	Weakness												
***N*** **= 788**	No (750)	Ref (1.000)			Ref (1.000)			Ref (1.000)			Ref (1.000)		
	Yes (38)	2.205 (1.146, 4.243)	5.61,1	0.018	2.098 (1.078, 4.083)	4.755,1	0.029	2.103 (1.068, 4.142)	4.625,1	0.032	2.080 (1.055, 4.104)	4.467,1	0.035
	**Slowness**												
	No (639)	Ref (1.000)			Ref (1.000)			Ref (1.000)			Ref (1.000)		
	Yes (149)	1.006 (0.687, 1.474)	0.001,1	0.975	1.047 (0.708, 1.547)	0.052,1	0.820	0.992 (0.667, 1.477)	0.002,1	0.969	0.998 (0.670, 1.487)	0.000,1	0.993
	**Weight loss**												
	No (722)	Ref (1.000)			Ref (1.000)			Ref (1.000)			Ref (1.000)		
	Yes (66)	0.986 (0.574, 1.693)	0.003,1	0.958	0.994 (0.575, 1.72)	0,1	0.984	1.017 (0.585, 1.77)	0.004,1	0.951	0.992 (0.568, 1.735)	0.001,1	0.979
	**Exhaustion**												
	No (656)	Ref (1.000)			Ref (1.000)			Ref (1.000)			Ref (1.000)		
	Yes (132)	1.853 (1.264, 2.716)	9.997,1	0.002	1.961 (1.326, 2.898)	11.394,1	0.001	1.936 (1.302, 2.877)	10.674,1	0.001	1.928 (1.297, 2.867)	10.542,1	0.001
	**Low activity**												
	No (698)	Ref (1.000)			Ref (1.000)			Ref (1.000)			Ref (1.000)		
	Yes (90)	1.478 (0.941, 2.32)	2.878,1	0.090	1.618 (1.016, 2.576)	4.113,1	0.043	1.566 (0.979, 2.505)	3.495,1	0.062	1.582 (0.988, 2.533)	3.652,1	0.056

*Model 1, unadjusted; Model 2, adjusted for age, sex, educational levels, marital status, live place; Model 3, adjusted for age, sex, educational levels, marital status, live place, current smoking, alcohol drinking, activities, chronic diseases; Model 4, adjusted for age, sex, educational levels, marital status, live place, current smoking, alcohol drinking, activities, chronic diseases, BMI*.

## Discussion

Previous studies have reported differences in the relationship between frailty and the incidence of depressive symptoms. Furthermore, the results in the association among the mid-aged and elderly in China have been sparse. Our study describes the cross-sectional and longitudinal associations between pre-frailty/frailty/components of frailty and depressive symptoms. Firstly, it is confirmed that pre-frailty/frailty/components of frailty (exhaust, weakness, weight loss, and slowness) at baseline was related to depressive symptoms; Secondly, pre-frailty/frailty at baseline was not significantly associated with the onset of depressive symptoms after 2 years of follow-up. Among specific criteria, slowness was a significant independent predictor of future depressive symptoms. Lastly, pre-frailty/frailty at baseline was significantly associated with the onset of depressive symptoms after 4 years of follow-up. Among specific criteria, weakness and exhaustion were significant independent predictors of future depressive symptoms.

Although several meta-analysis studies (11–15) included cohort studies and cross-sectional studies and highlighted the potential adverse effect of frailty on depression, no considerations of the components effect of frailty on depression over several years in different studies were made. Moreover, the meta-analysis only included Western participants, and no considerations of other ethnicities, such as Asians. Thus, further studies among the middle-aged and elderly in Asian nations are needed to identify whether the relationship between frailty and incidence of depression in Asian participants. Several findings from longitudinal studies have found that factors similar to components of frailty, such as physical activity (32, 33), fatigue (34–36) and mobility impairment (37) appear to increase the risk for developing depressive symptoms in the older adult. Findings from the present longitudinal data in our study indicate that preferability/frailty is associated with increased risk of incident depressive symptoms after 4 years of follow-up in the middle-aged and elderly aged 45–96 years was in line with the previous studies conducted in the United Kingdom (22), Japan (38), Italians (39), and mainland China (22, 40–42), although the measurements of the frailty phenotype, the population and the years of follow-up were different. Interestingly, we did not find a significant association between baseline preferability/frailty and the longitudinal onset of depressive symptoms after 2 years of follow-up. The phenomenon could be explained by the cumulative effect, which showed a significant association between preferability/frailty and the longitudinal onset of depressive symptoms over long-term exposure (4 years of follow-up).

With regard to the components of frailty, we found slowness was related to increased risk of incident depressive symptoms after 2 years of follow-up in older adults without baseline depressive symptoms. Moreover, weakness and exhaustion were significantly associated with the onset of depressive symptoms after 4 years of follow-up. However, the findings are partly in accordance with previous studies. Veronese et al. (23) using data from 4,077 representative of people living in England aged 50 years and over, found that slowness (slow gait speed) considered alone predicted depression. Collard et al. (39) launched another similar study to discuss the association and found that Low physical activity was associated with incident depressive symptoms. Chu et al. (42) conducted a population-based cohort study including 1,788 older adults aged 70–84 years in Rugao, Jiangsu Province, China and found that weakness (lower grip strength) was associated with incident depressive symptoms. Several hypotheses could be used to explain the differences between our study and the previously mentioned studies in the literature. First, methodological differences (evaluation tools for depressive symptoms and frailty, length of follow up and confounders) reported in these studies may play an important role. For example, 10-item Epidemiologic Studies-Depression scale for evaluating depressive symptoms was used in our study, but 20-item Epidemiologic Studies-Depression scale and 15-item Geriatric Depression Scale (GDS-15) in other studies (23, 39, 42). Second, the different results reflect that the association may be influenced by cultural background. Furthermore, it is likely adaption of revised Fried's criteria (5) used in our study may influence our result. The mechanisms underline the relationship between frailty and incidence of depressive symptoms is still unknown. Frail individuals may develop depressive symptoms through impaired function, lack of social community, and lower physical activity (42). Several hypotheses could explain the significant relationship between slowness and the onset of depressive symptoms (2 years of follow-up). Firstly, slowness might be an early marker of a depressed mood (43). Secondly, slowness and depressive symptoms shared some risk and pathogenic factor that might affect the onset of depressive symptoms (23). Thirdly, individuals with slowness might be socially isolated, and could be more depressive symptoms (44). Finally, individuals with slowness might have lower physical activity, and might increase the risk of future depressive symptoms (45). However, weakness and exhaustion were significantly associated with the onset of depressive symptoms after 4 years of follow-up. Long exposure to the frailty, individuals with weakness and exhaustion may impair posterior aspects of brain (40, 46), and increased low-grade inflammation such as C-reactive protein (CRP), interleukin-6 (IL-6), or tumor necrosis factor-α(TNF-α), and mediated the risk of depressive symptoms. Interventions designed to prevent depressive symptoms may be useful in reducing frailty among middle-aged and older adults.

## Strengths and Limitations of the Study

Our study has several strengths. The study was based on a nationwide population-based cohort study, which included participants aged ≥45 years. It compared the effect of frailty and its components across two different intervals on the depressive symptom. Previous studies used only a set single interval to identify the relationship between frailty and depressive symptom. It helped us to understand the short- and long-term effects of frailty on the incidence of depressive symptoms. Several limitations in our study should be noted. The depressive symptom was self-reported in the three waves when it was subjectively measured. This may have a reporting bias. It is known that people tend to underreport their mental illness in the research. Many participants were excluded for the missing data, and further research should focus more on a set of complete material.

## Conclusions

Among middle-aged and older adults, frailty, pre-frailty did not predict depressive symptoms during 2 years of follow-up, when accounting for the potential confounders, slowness considered alone predicted depressive symptoms. Additionally, frailty, pre-frailty predicted depressive symptoms during 4 years of follow-up, when accounting for the potential confounders, weakness and exhaustion considered alone predicted depressive symptoms.

## Data Availability Statement

The raw data supporting the conclusions of this article will be made available by the authors, without undue reservation.

## Ethics Statement

Approval for this study was given by the Medical Ethics Committee of Wannan Medical College (Approval Number 2021–3). The patients/participants provided their written informed consent to participate in this study.

## Author Contributions

HL and LZ: conceived and designed the research. LZ: wrote the article and analyzed the data. HL, LZ, XY, GX, J-lL, L-lG, LY, CW, TY, DZ, HW, JL, YLe, LS, XL, YH, HC, ML, and YLi: revised the article. All authors reviewed the manuscript. All authors contributed to the article and approved the submitted version.

## Funding

CHARLS was supported by the NSFC (70910107022 and 71130002) and National Institute on Aging (R03-TW008358-01 and R01-AG037031-03S1) and World Bank (7159234), and the Support Program for Outstanding Young Talents from the Universities and Colleges of Anhui Province for LZ (gxyqZD2021118).

## Conflict of Interest

The authors declare that the research was conducted in the absence of any commercial or financial relationships that could be construed as a potential conflict of interest.

## Publisher's Note

All claims expressed in this article are solely those of the authors and do not necessarily represent those of their affiliated organizations, or those of the publisher, the editors and the reviewers. Any product that may be evaluated in this article, or claim that may be made by its manufacturer, is not guaranteed or endorsed by the publisher.

## Abbreviations

CHARLS: China Health and Retirement Longitudinal Study
CAPI: Computer-Aided Personal Interview
HRs: Hazard Ratios
OR: Odds Ratio
BMI: Body Mass Index
M: Mean
SD: Standard Deviation
CIs: Confidence Intervals
CESD: Chinese version of the Center for Epidemiologic Studies-Depression scale
CRP: C-Reactive Protein
IL-6: Interleukin-6
TNF-α: Tumor Necrosis Factor-α
GDS-15: Geriatric Depression Scale
NSFC: The National Natural Science Foundation of China
NIA: National Institute on Aging
WB: World Bank.

## References

[1] Zhang L Liu K Li H Li D Chen Z Zhang LL. Relationship between body mass index and depressive symptoms: the “fat and jolly” hypothesis for the middle-aged and elderly in China. BMC Public Health. (2016) 16:1201. 10.1186/s12889-016-3864-5 27894296 PMC5126817

[2] da Silva SA Scazufca M Menezes PR. Population impact of depression on functional disability in elderly: results from “São Paulo ageing & health study” (SPAH). Eur Arch Psychiatry Clin Neurosci. (2013) 263:153–8. 10.1007/s00406-012-0345-4 22872105

[3] Kang HJ Bae KY Kim SW Shin HY Shin IS Yoon JS. Impact of anxiety and depression on physical health condition and disability in an elderly Korean population. Psychiatry Investig. (2017) 14:240–8. 10.4306/pi.2017.14.3.240 28539942 PMC5440426

[4] Thinuan P Siviroj P Lerttrakarnnon P Lorga T. Prevalence and potential predictors of frailty among community-dwelling older persons in northern thailand: a cross-sectional study. Int J Environ Res Public Health. (2020) 17:4077. 10.3390/ijerph17114077 32521642 PMC7312471

[5] Fried LP Tangen CM Walston J Newman AB Hirsch C Gottdiener J. Frailty in older adults: evidence for a phenotype. J Gerontol A Biol Sci Med Sci. (2001) 56:M146–56. 10.1093/gerona/56.3.M146 11253156

[6] Collard RM Boter H Schoevers RA Oude Voshaar RC. Prevalence of frailty in community-dwelling older persons: a systematic review. J Am Geriatr Soc. (2012) 60:1487–92. 10.1111/j.1532-5415.2012.04054.x 22881367

[7] O'Caoimh R Sezgin D O'Donovan MR Molloy DW Clegg A Rockwood K. Prevalence of frailty in 62 countries across the world: a systematic review and meta-analysis of population-level studies. Age Ageing. (2021) 50:96–104. 10.1093/ageing/afaa219 33068107

[8] Santos-Eggimann B Cuénoud P Spagnoli J Junod J. Prevalence of frailty in middle-aged and older community-dwelling Europeans living in 10 countries. J Gerontol A Biol Sci Med Sci. (2009) 64:675–81. 10.1093/gerona/glp012 19276189 PMC2800805

[9] Guessous I Luthi JC Bowling CB Theler JM Paccaud F Gaspoz JM. Prevalence of frailty indicators and association with socioeconomic status in middle-aged and older adults in a swiss region with universal health insurance coverage: a population-based cross-sectional study. J Aging Res. (2014) 2014:198603. 10.1155/2014/198603 25405033 PMC4227447

[10] Bhatt PP Sheth MS. Prevalence of frailty in middle-aged diabetic population of ahmedabad: a cross-sectional study. Indian J Endocrinol Metab. (2021) 25:254–5. 10.4103/ijem.ijem_193_21 34760684 PMC8547396

[11] Chu W Chang SF Ho HY Lin HC. The relationship between depression and frailty in community-dwelling older people: a systematic review and meta-analysis of 84,351 older adults. J Nurs Scholarsh. (2019) 51:547–59. 10.1111/jnu.12501 31328878

[12] Soysal P Veronese N Thompson T Kahl KG Fernandes BS Prina AM. Relationship between depression and frailty in older adults: a systematic review and meta-analysis. Ageing Res Rev. (2017) 36:78–87. 10.1016/j.arr.2017.03.005 28366616

[13] Mezuk B Edwards L Lohman M Choi M Lapane K. Depression and frailty in later life: a synthetic review. Int J Geriatr Psychiatry. (2012) 27:879–92. 10.1002/gps.2807 21984056 PMC3276735

[14] Buigues C Padilla-Sanchez C Garrido JF Navarro-Martinez R Ruiz-Ros V Cauli O. The relationship between depression and frailty syndrome: a systematic review. Aging Ment Health. (2015) 19:762–72. 10.1080/13607863.2014.967174 25319638

[15] Vaughan L Corbin AL Goveas JS. Depression and frailty in later life: a systematic review. Clin Interv Aging. (2015) 10:1947–58. 10.2147/CIA.S69632 26719681 PMC4687619

[16] Zhang J Norvilitis JM. Measuring Chinese psychological well-being with Western developed instruments. J Pers Assess. (2002) 79:492–511. 10.1207/S15327752JPA7903_06 12511017

[17] Wang JN Sun W Chi TS Wu H Wang L. Prevalence and associated factors of depressive symptoms among Chinese doctors: a cross-sectional survey. Int Arch Occup Environ Health. (2010) 83:905–11. 10.1007/s00420-010-0508-4 20112108

[18] Liu L Chang Y Fu J Wang J Wang L. The mediating role of psychological capital on the association between occupational stress and depressive symptoms among Chinese physicians: a cross-sectional study. BMC Public Health. (2012) 12:219. 10.1186/1471-2458-12-219 22436106 PMC3410796

[19] Yu J Li J Cuijpers P Wu S Wu Z. Prevalence and correlates of depressive symptoms in Chinese older adults: a population-based study. Int J Geriatr Psychiatry. (2012) 27:305–12. 10.1002/gps.2721 21538538

[20] Steptoe A Breeze E Banks J Nazroo J. Cohort profile: the english longitudinal study of ageing. Int J Epidemiol. (2013) 42:1640–8. 10.1093/ije/dys168 23143611 PMC3900867

[21] Theou O Cann L Blodgett J Wallace LM Brothers TD Rockwood K. Modifications to the frailty phenotype criteria: systematic review of the current literature and investigation of 262 frailty phenotypes in the survey of health, ageing, and retirement in Europe. Ageing Res Rev. (2015) 21:78–94. 10.1016/j.arr.2015.04.001 25846451

[22] Xue G Yang Y Yu S Fu P Xu W Li YX. Incidence of frailty among community-dwelling older adults: a nationally representative profile in China. J Clin Nurs. (2019) 19:378. 10.1186/s12877-019-1393-7 31888498 PMC6937935

[23] Veronese N Solmi M Maggi S Noale M Sergi G Manzato E. Frailty and incident depression in community-dwelling older people: results from the ELSA study. Int J Geriatr Psychiatry. (2017) 32:e141–e9. 10.1002/gps.4673 28195361

[24] Zhang L Li JL Zhang LL Guo LL Li H Li D. Association and interaction analysis of body mass index and triglycerides level with blood pressure in elderly individuals in China. Biomed Res Int. (2018) 2018:8934534. 10.1155/2018/8934534 30596101 PMC6282155

[25] Zhang L Li JL Zhang LL Guo LL Li H Yan W. Relationship between adiposity parameters and cognition: the “fat and jolly” hypothesis in middle-aged and elderly people in China. Medicine. (2019) 98:e14747. 10.1097/MD.0000000000014747 30855469 PMC6417533

[26] Zhang L Li JL Zhang LL Guo LL Li H Li D. Body mass index and serum uric acid level: Individual and combined effects on blood pressure in middle-aged and older individuals in China. Medicine. (2020) 99:e19418. 10.1097/MD.0000000000019418 32118796 PMC7478523

[27] Chang HH Yen ST. Association between obesity and depression: evidence from a longitudinal sample of the elderly in Taiwan. Aging Ment Health. (2012) 16:173–80. 10.1080/13607863.2011.605053 21861766

[28] Zhang L Li JL Zhang LL Guo LL Li H Li D. No association between C-reactive protein and depressive symptoms among the middle-aged and elderly in China: evidence from the China health and retirement longitudinal study. Medicine. (2018) 97:e12352. 10.1097/MD.0000000000012352 30235693 PMC6160211

[29] Zhang L Li JL Guo LL Li H Li D Xu G. The interaction between serum uric acid and triglycerides level on blood pressure in middle-aged and elderly individuals in China: result from a large national cohort study. BMC Cardiovasc Disord. (2020) 20:174. 10.1186/s12872-020-01468-3 32293295 PMC7160924

[30] Zhang L Yang L Wang C Yuan T Zhang D Wei H. Combined effect of famine exposure and obesity parameters on hypertension in the midaged and older adult: a population-based cross-sectional study. Biomed Res Int. (2021) 2021:5594718. 10.1155/2021/5594718 34604385 PMC8486537

[31] Zhang L Yang L Wang C Yuan T Zhang D Wei H. Individual and combined association analysis of famine exposure and serum uric acid with hypertension in the mid-aged and older adult: a population-based cross-sectional study. Biomed Res Int. (2021) 21:420. 10.1186/s12872-021-02230-z 34488649 PMC8420034

[32] Schuch FB Vancampfort D Firth J Rosenbaum S Ward PB Silva ES. Physical activity and incident depression: a meta-analysis of prospective cohort studies. Depress Anxiety. (2018) 175:631–48. 10.1176/appi.ajp.2018.17111194 29690792

[33] Choi KW Zheutlin AB Karlson RA Wang MJ. Physical activity offsets genetic risk for incident depression assessed via electronic health records in a biobank cohort study. Depress Anxiety. (2020) 37:106–14. 10.1002/da.22967 31689000 PMC7905987

[34] Bohman H Jonsson U Päären A von Knorring L Olsson G von Knorring AL. Prognostic significance of functional somatic symptoms in adolescence: a 15-year community-based follow-up study of adolescents with depression compared with healthy peers. J Korean Med Sci. (2012) 12:90. 10.1186/1471-244X-12-90 22839681 PMC3439696

[35] Yu DS Lee DT. Do medically unexplained somatic symptoms predict depression in older Chinese? Int J Geriatr Psychiatry. (2012) 27:119–26. 10.1002/gps.2692 22223144

[36] Jeon SW Yoon SY. Do somatic symptoms predict the severity of depression? a validation study of the Korean version of the depression and somatic symptoms scale. J Korean Med Sci. (2016) 31:2002–9. 10.3346/jkms.2016.31.12.2002 27822942 PMC5102867

[37] Chan LLY Okubo Y Brodie MA Lord SR. Mobility performance predicts incident depression: a systematic review and meta-analysis. Exp Gerontol. (2020) 142:111116. 10.1016/j.exger.2020.111116 33086078

[38] Makizako H Shimada H Doi T Yoshida D Anan Y Tsutsumimoto K. Physical frailty predicts incident depressive symptoms in elderly people: prospective findings from the obu study of health promotion for the elderly. J Am Med Dir Assoc. (2015) 16:194–9. 10.1016/j.jamda.2014.08.017 25307294

[39] Collard RM Comijs HC Naarding P Penninx BW Milaneschi Y Ferrucci L. Frailty as a predictor of the incidence and course of depressed mood. J Am Med Dir Assoc. (2015) 16:509–14. 10.1016/j.jamda.2015.01.088 25737263 PMC5127267

[40] Feng L Nyunt MS Feng L Yap KB Ng TP. Frailty predicts new and persistent depressive symptoms among community-dwelling older adults: findings from Singapore longitudinal aging study. J Am Med Direct Assoc. (2014) 15:76. 10.1016/j.jamda.2013.10.001 24314697

[41] Zhang N Shi GP Wang Y Chu XF Wang ZD Shi JM. Depressive symptoms are associated with incident frailty in a Chinese population: the rugao longevity and aging study. Aging Clin Exp Res. (2020) 32:2297–302. 10.1007/s40520-019-01409-x 31786744

[42] Chu XF Zhang N Shi GP Wang Y Wang ZD Guo JH. Frailty and incident depressive symptoms in a Chinese sample: the rugao longevity and ageing study. Psychogeriatrics. (2020) 20:691–8. 10.1111/psyg.12565 32558008

[43] Tuerk C Zhang H Sachdev P Lord SR Brodaty H Wen W. Regional gray matter volumes are related to concern about falling in older people: a voxel-based morphometric study. J Gerontol A Biol Sci Med Sci. (2016) 71:138–44. 10.1093/gerona/glu242 25645388

[44] Singh A Misra N. Loneliness, depression and sociability in old age. Ind Psychiatry J. (2009) 18:51–5. 10.4103/0972-6748.57861 21234164 PMC3016701

[45] Vancampfort D Stubbs B. Physical activity and metabolic disease among people with affective disorders: prevention, management and implementation. J Affect Disord. (2017) 224:87–94. 10.1016/j.jad.2016.07.042 27519365

[46] Katz IR. Depression and frailty: the need for multidisciplinary research. Am J Geriatr Psychiatry. (2004) 12:1–6. 10.1176/appi.ajgp.12.1.1 14729553

